# Translational Potential of a Contrast Agent for FGS Applications in pNETs

**DOI:** 10.1007/s11307-024-01894-1

**Published:** 2024-01-24

**Authors:** Solmaz AghaAmiri, Jeannelyn S. Estrella, Servando Hernandez Vargas, Mark W. Hurd, Sukhen C. Ghosh, Ali Azhdarinia, Naruhiko Ikoma

**Affiliations:** 1 The Brown Foundation Institute of Molecular Medicine, McGovern Medical School, The University of Texas Health Science Center at Houston, Houston, TX 77054 USA; 2 Department of Anatomic Pathology, The University of Texas MD Anderson Cancer Center, Houston, TX 77030 USA; 3 Sheikh Ahmed Center for Pancreatic Cancer Research, The University of Texas MD Anderson Cancer Center, Houston, TX 77030 USA; 4 Department of Surgical Oncology, The University of Texas MD Anderson Cancer Center, Houston, TX 77030 USA

**Keywords:** Neuroendocrine tumors, FGS, SSTR2, pNET

The critical need for accurate tumor detection during oncologic surgery has led to the development of fluorescent contrast agents that target cancerous tissues. This technique, known as fluorescence-guided surgery (FGS), typically involves systemic administration of a fluorescent compound that contains a tumor-targeting moiety and is imaged in the near-infrared spectral range in order to maximize image contrast and diagnostic accuracy [1]. In 2021, the folic acid analog OTL-38 (CYTALUX®) became the first clinically approved FGS agent to target tumors via an active, receptor-mediated process. As shown in a randomized phase 3 study, lesions in ovarian cancer patients undergoing cytoreductive surgery were more effectively identified with FGS than with standard-of-care methods [2]. Findings from this landmark study not only demonstrated the value of FGS in ovarian cancer patients but also set an important translational precedent for applying emerging FGS agents to other cancers.

Pancreatic neuroendocrine tumors (pNETs) are a type of cancer where molecularly driven FGS could significantly improve surgical outcomes. Accurate intraoperative localization of primary and metastatic pNETs is challenging, particularly for small, multifocal lesions, and leads to incomplete resection in 15–45% of cases [3]. Also, unnecessarily extended resections can cause subsequent pancreatic insufficiency: about 40% of patients who undergo pNET resection experience endocrine insufficiency in the form of de novo diabetes [4, 5], and 20–30% develop exocrine insufficiency and impaired digestion [5, 6]. Given the long life expectancy of pNET patients, preservation of pancreatic function and complete resection of lesions are of paramount importance [7], thus, accurate intraoperative tumor localization is critical.

Given the overexpression of somatostatin receptor subtype-2 (SSTR2) in the vast majority of pNETs, we selected the clinically approved SSTR2-targeted radiopharmaceutical ^68^Ga-DOTA-TOC as a foundation for developing a fluorescent counterpart and produced a first-generation agent that showed SSTR2-mediated targeting in cancer cells and multiple *in vivo* tumor models [8]. To further increase image contrast, we developed an optimized second-generation agent, MMC(FNIR-Tag)-TOC, that contains a charge-balanced fluorophore (FNIR-Tag, λ_abs_ = 772 and λ_em_ = 788 nm). Evaluation in translationally relevant tumor models revealed that MMC(FNIR-Tag)-TOC has superior targeting specificity and pharmacokinetics compared to our initial agent, which resulted in markedly higher contrast between tumor and normal tissue [9]. Accordingly, we identified MMC(FNIR-Tag)-TOC as the lead compound for clinical development.

**Fig. 1:**
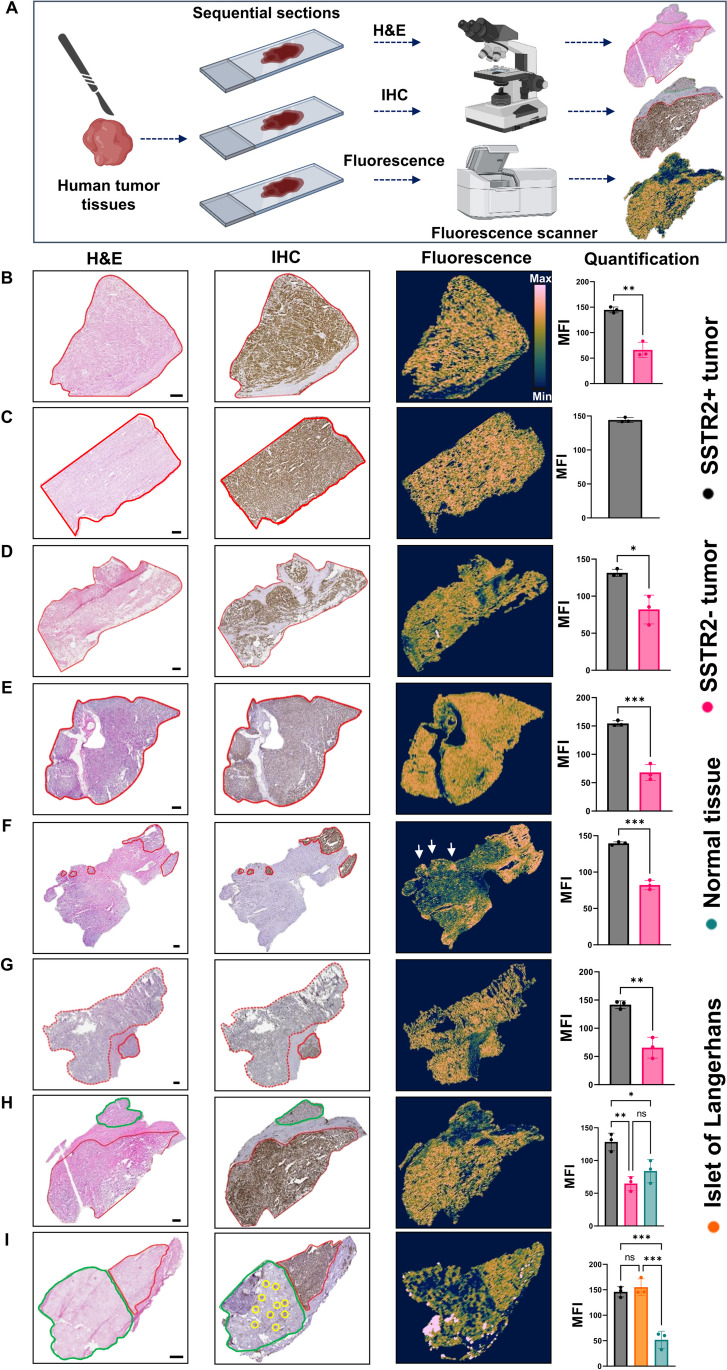
*Ex vivo* characterization of SSTR2-targeted fluorescence imaging in pNET biospecimens. **A** Schematic representation of biospecimen staining and imaging procedures. **B**–**I** Pathological assessments of pNET tissue sections (*n* = 8) as determined by H&E (morphological topography) and SSTR2 IHC (molecular topography; brown staining shows SSTR2 distribution and intensity) were qualitatively compared to the fluorescence map (imaging topography) afforded by the SSTR2-targeting FGS agent MMC(FNIR-Tag)-TOC. Quantitative analysis was in agreement with the qualitative observation, showing significantly higher fluorescence intensity in SSTR2-positive areas than in SSTR2-negative areas. Solid/dashed red and green lines indicate tumor and normal regions, respectively, as determined by H&E. White arrows indicate small, multifocal SSTR2-positive regions detected by the FGS agent, which were confirmed by IHC. Yellow circles indicate islets of Langerhans. The color map applied in the fluorescence panel is batlow [10]. Scale bar: 500 μm *, *P* ≤ 0.01; **, *P* ≤ 0.001; ***, *P* ≤ 0.0001

To bridge the gap between preclinical and clinical development of MMC(FNIR-Tag)-TOC, we implemented a fluorescence-guided pathology strategy to (i) examine the binding of our agent to human tumors and (ii) investigate its potential use for delineation of surgical margins. We obtained whole tissue sections (n = 8) from patients with pNET who underwent curative-intent pancreatectomy from the Institutional Tissue Bank at The University of Texas MD Anderson Cancer Center after Institutional Review Board approval. The tissues were snap-frozen and serially sectioned into 5 µm frozen sections for *ex vivo* staining with MMC(FNIR-Tag)-TOC as described previously [8]. The stained sections were scanned for fluorescence signal using a near-infrared fluorescence imaging system (Odyssey, LI-COR). Sequential sections from each specimen were also stained with standard hematoxylin and eosin (H&E) and immunohistochemistry (IHC) for SSTR2 (ab134152, Abcam, 1:1000 dilution) to allow morphologic evaluation of tumor and normal tissues and to assess SSTR2 expression in the samples, respectively. We performed a qualitative analysis and evaluated the correlation between the fluorescence staining pattern and SSTR2 distribution in the tumor and surrounding normal areas of the sections. Additionally, we conducted a quantitative analysis to measure the fluorescence intensity of identically sized regions of interest (ROIs) in both tumor and adjacent normal tissues using ImageJ (Fig. 1A).

Six tissue sections contained tumor only (Fig. 1B–G, red area), and two samples contained both tumor (red area) and normal pancreas (green area) (Fig. 1H and I). SSTR2 IHC signal was positive in tumor regions, with strong and widespread staining intensity in all cases except one (Fig. 1G), where variable intensity was seen as indicated by areas of both strong (solid line) and weak (dashed line) staining in the tumor. As shown in Fig. 1H and I, SSTR2 IHC showed minimal background staining in pancreatic acinar cells (normal pancreas, marked green), while highlighting islets of Langerhans (Fig. 1I, yellow circles). Fluorescence imaging showed excellent co-localization of MMC(FNIR-Tag)-TOC with SSTR2-positive areas and small multifocal lesions (Fig. 1F, approximate size: 600–900 μm) that were within the tumor boundary, as confirmed by H&E and IHC staining. Figure 1I shows the presence of punctate fluorescence in the normal pancreas, which correlated with islets of Langerhans (approximate size: 200 μm) and was also confirmed by H&E and IHC staining for SSTR2. Quantitative analysis of the sections was consistent with the qualitative observations and revealed higher mean fluorescent intensity (MFI) values in SSTR2-positive areas compared to SSTR2-negative areas (Fig. 1 right panel).

This proof-of-concept study confirms that the receptor-mediated binding of MMC(FNIR-Tag)-TOC observed in our previous animal studies extends to human tumors. This study also reveals potential limitations of our approach in clinical use. First, since our agent relies solely on SSTR2 expression in the tumor, variability of SSTR2 expression in pNETs and physiologic SSTR2 expression in normal pancreatic tissue can confound the interpretation of imaging findings. Thus, preoperative patient selection with ^68^Ga-DOTATATE PET will play an important role in evaluating adequate SSTR2 expression in pNET lesions. Another limitation is the need for standardization of the fluorescence imaging parameters. We adjusted the gain of the imaging system in order to optimize the fluorescence signal intensity in each specimen and maximize tumor-background contrast. In future applications of our fluorescence-guided pathology approach, such as determining surgical margins, further standardization of the imaging settings will be needed to quantify the absolute intensity of the fluorescence signal produced by the tumor and will require validation in human specimens in a clinical trial.

In the envisioned clinical scenario, injection of MMC(FNIR-Tag)-TOC before curative-intent surgical resection of pNETs will enable two primary applications. First, intraoperative guidance for tumor localization will be possible due to the ability of the agent to specifically “light up” pNETs with fluorescence imaging (*e.g.*, via FireFly imaging system in the *daVinci* robotic surgery platform), allowing surgeons to detect primary and metastatic lesions (including positive lymph nodes) and achieve optimal surgical resections. Second, margin status will be assessed by (i) intraoperative imaging of the surgical bed after resection to confirm the lack of residual fluorescence signal, (ii) back-table fluorescence imaging of the surgical specimen to show adequate distance between the tumor and the surgical margin, and (iii) fluorescence-guided pathology to directly evaluate the surgical margins. Since the tissue is already “stained” via preoperative systemic injection of MMC(FNIR-Tag)-TOC, fluorescence-guidance at the mesoscopic level could facilitate pathological analysis and interpretation. As described above, the timing and dose of the injection will have to be carefully investigated in a clinical trial to achieve optimal tumor-to-background contrast, with standardized imaging settings for intraoperative imaging as well as for pathological assessment.

In summary, our findings show that MMC(FNIR-Tag)-TOC has high specificity for pNET tissues. The observed fluorescence signal was largely correlated with SSTR2 IHC staining and accurately represented pNET tumor extension, which suggests strong utility for fluorescence-guided surgical applications. Accordingly, we plan to extend our research into the clinical arena and initiate a first-in-human trial to investigate the feasibility of using MMC(FNIR-Tag)-TOC for FGS in patients with pNETs.
